# Dynamic Characterization of Microstructured Materials Made of Hexagonal-Shape Particles with Elastic Interfaces

**DOI:** 10.3390/nano11071781

**Published:** 2021-07-08

**Authors:** Marco Colatosti, Nicholas Fantuzzi, Patrizia Trovalusci

**Affiliations:** 1 DISG Department, Sapienza University of Rome, via A. Gramsci 53, 00197 Rome, Italy; marco.colatosti@uniroma1.it (M.C.); patrizia.trovalusci@uniroma1.it (P.T.); 2 DICAM Department, University of Bologna, Viale del Risorgimento 2, 40136 Bologna, Italy

**Keywords:** composite materials, cosserat theory, dynamics, finite element method, hexagonal shaped particles

## Abstract

This work aims to present the dynamic character of microstructured materials made of hexagonal-shape particles interacting with elastic interfaces. Several hexagonal shapes are analyzed to underline the different constitutive behavior of each texture. The mechanical behavior at the macro scale is analyzed by considering a discrete model assumed as a benchmark of the problem and it is compared to a homogenized micropolar model as well as a classical one. The advantages of the micropolar description with respect to the classical one are highlighted when internal lengths and anisotropies of microstuctured materials are taken into consideration. Comparisons are presented in terms of natural frequencies and modes of vibrations.

## 1. Introduction

Particle composites are a class of materials which present an internal microstructure constituted by particles and interfaces: ceramic and metal composites, poly-crystals, masonry, porous rocks are some examples of media characterized by this peculiarity. In order to describe the macroscopic response of these materials is fundamental to detect the influences of the microscopic scale: a possible approach to study the mechanical response is to realize a discrete model of the microstructure. However, an approach like this, results to be computationally cumbersome [1,2,3], both for the microstructure and for the high number of the degrees of freedom. An alternative approach is to homogenize particle composites in an equivalent continuum that takes into account all the mechanical aspects of the microstructure. This strategy is faster and computationally less expensive [4], nevertheless the selection of the homogenization procedure is a challenging task, mostly because it requires the choice of the proper macroscopic continuum that is able to preserve memory of the microstructure not only in terms of shape and arrangements of the elements but also of their size, in problems where the internal length effects are not negligible [5,6].

It is recognized that the classical continuum is not always suitable for capturing the macroscopic behaviour of these composite materials [7]. Some continuum theories have non-local character for the presence of the internal length, as the distance between particles in a discrete structure, the grain or cell size, the correlation radius of at-a-distance force, or due to spatial dispersion properties, in fact there may be a dependence of the wave velocities on wavelength or frequency [8,9,10].

Starting from this circumstance different models have been presented as the strain gradient [11,12,13], and micropolar continua, that can be considered non-local models of implicit type [4,14,15,16,17,18], one of the peculiarities of the latter is that they include additional degrees of freedom [19].

One of the cause of great interest by researchers to apply these non-local theories is to properly describe the buckling and dynamical behaviour of composite materials and nanomaterials, such as nanoplates, nanorods [20], nanobeams, composite plates, which are widely used in many industrial fields; the strain gradient theory is largely adopted [21,22,23,24,25,26,27,28], as well as the modified strain gradient theory, [29,30,31,32,33,34]. Finally, many works concerning the study of the elastic [35,36], viscoelastic [37] and elastoplastic [38] behavior of composite materials are based on different homogenization approaches [39,40,41].

As regards the micropolar continuum, it takes into account the strain measure of the microrotation, which makes a contribution in anisotropic media [42]. Moreover, it is useful to emphasize the effects of the additional strain measure of the so-called relative rotation, defined as the difference between microrotation and macrorotation, the local rigid rotation, corresponding to the skew-symmetric part of the displacement gradient [17,43].

Furthermore, several studies on the dynamics of particle materials are present in the literature [44], in particular for hexagonal lattice systems, [45,46,47], materials with periodic hexagonal microstructure [48,49], chiral materials [50], granular matter [51] and polymeric composites [52]. Consequently, it is of interest to study materials of this type as continuous models, and the Cosserat theory is widely applied to study the dynamic behaviour of media with internal microstrucuture, such as: granular materials [53], plates [54] and shells [55], composite materials [56], masonry structures [57,58] and to investigate dispersive properties [59,60].

In this paper, the aim is to characterize the dynamic behavior of microstructured materials, in particular materials endowed of particles, with three different hexagonal shapes, and thin elastic interfaces modelled as a Cosserat continuum and to highlight the advantages in comparison with the Cauchy continuum [61,62,63,64], whereas a discrete model is assumed as a benchmark characterized by rigid blocks and linear elastic springs at the interfaces [65]. To allow this, the approach, used in this study, consists in the description of a continuum model and of a complex lattice model which are linked through the field variables via an energy equivalence criterion [5,6,66,67,68,69].

The paper is structured in this way: in Section 2 a short introduction about the main micropolar continuum aspects is presented, in Section 3 details about the representative volume element and materials constitutive properties are discussed; in Section 4 the numerical implementation of models is discussed and at last, in Section 5, free vibration simulations [70,71] for a comparison between the discrete model, assumed as benchmark, and the micropolar and classical continuum are reported and finally the most important aspects will be highlighted.

## 2. Micropolar Continuum

The present work refers to two-dimensional (2D) media and each material particle has three degrees of freedom: $u_{1}$ and $u_{2}$ are the displacement components and ω is the micro-rotation. The term ω, is different from the macro-rotation θ, defined as the skew-symmetric part of the gradient of displacement. The displacement vector is $u^{⊤}=\left[\begin{array}{ccc} u_{1} & u_{2} & \omega \end{array}\right]$, and the strain vector is: $\varepsilon^{⊤}=\left[\begin{array}{cccccc} \varepsilon_{11} & \varepsilon_{22} & \varepsilon_{12} & \varepsilon_{21} & \kappa_{1} & \kappa_{2} \end{array}\right]$, where $\varepsilon_{ij}$ are the normal and shear strains and the microcurvatures are indicated by $\kappa_{1}$ and $\kappa_{2}$. Differently from the classical continuum the strain components are not reciprocal $\varepsilon_{12}\neq \varepsilon_{21}$. The stress vector is represented as: $\sigma^{⊤}=\left[\begin{array}{cccccc} \sigma_{11} & \sigma_{22} & \sigma_{12} & \sigma_{21} & \mu_{1} & \mu_{2} \end{array}\right]$ where $\sigma_{ij}$ for i,j=1,2 represents the normal and shear stress components and $\mu_{1}$, $\mu_{2}$ are the microcouples. The shear stress components are not reciprocal, $\sigma_{12}\neq \sigma_{21}$ and the couple stress components $\mu_{1}$, $\mu_{2}$ have to be introduced in order to satisfy the moment equilibrium of the micropolar body.

In matrix form, the kinematic compatibility relation is:

(1) ε=Du

where the operator D is:

(2) $$D^{⊤}=\left[\begin{array}{cccccc} \frac{\partial }{\partial x_{1}} & 0 & \frac{\partial }{\partial x_{2}} & 0 & 0 & 0\\ 0 & \frac{\partial }{\partial x_{2}} & 0 & \frac{\partial }{\partial x_{1}} & 0 & 0\\ 0 & 0 & 1 & -1 & \frac{\partial }{\partial x_{1}} & \frac{\partial }{\partial x_{2}} \end{array}\right]$$

The variation of internal work can be written as:

(3) $$\delta U=\int_{V}\delta \varepsilon^{⊤}\sigma \;dV=h\int_{A}\delta u^{⊤}D^{⊤}\;\sigma \;dA$$

where *h* is the thickness of the present 2D solid which will be considered as unitary. The kinetic energy is:

(4) $$\delta K=\int_{V}\rho \delta {\dot{u}}^{⊤}\dot{u}\;dV=\int_{A}\delta {\dot{u}}^{⊤}m\dot{u}\;dA=-\int_{A}\delta u^{⊤}m\ddot{u}\;dA$$

where m is the equivalent mass matrix defined as:

(5) $$m=\left[\begin{array}{ccc} \rho h & 0 & 0\\ 0 & \rho h & 0\\ 0 & 0 & \rho J_{c} \end{array}\right]$$

where ρ is the material density and $J_{c}$ represents the rotary inertia of the material point. Using the Hamilton’s principle (by neglecting external actions since only free vibrations will be here considered) the following equation is carried out:

(6) $$\delta \int_{t_{1}}^{t_{2}}\left(K-U\right)\;dt=0$$

considering the previous expressions:

(7) $$\int_{t_{1}}^{t_{2}}\int_{A}\delta u^{⊤}\left(m\ddot{u}+D^{⊤}\;\sigma \right)dA\;dt=0$$

The micropolar anisotropic constitutive equation takes the form:

(8) σ=Cε

where:

(9) $$C=\left[\begin{array}{cccccc} A_{1111} & A_{1122} & A_{1112} & A_{1121} & B_{111} & B_{112}\\ \; & A_{2222} & A_{2212} & A_{2221} & B_{221} & B_{222}\\ \; & \; & A_{1212} & A_{1221} & B_{121} & B_{122}\\ \; & \; & & A_{2121} & B_{211} & B_{212}\\ \; & \; & & \; & D_{11} & D_{12}\\ sym & \; & & \; & & D_{22} \end{array}\right]$$

By considering hyperelastic materials, the constitutive matrix is symmetric (C∈Sym): in particular $A_{ijhk}=A_{hkij}$; $B_{ijh}=B_{hij}$; $D_{ij}=D_{ji}$ [5]. Accounting for the constitutive equations, the Hamilton principle for free vibrations can be formulated:

(10) $$\int_{t_{1}}^{t_{2}}\int_{A}\delta u^{⊤}\left(m\ddot{u}+D^{⊤}\;CDu\right)dA\;dt=0$$

## 3. Reference Volume Element

The constitutive matrix (9) can be carried out by homogenization according to a multi-scale approach [5]. It has been recently demonstrated that a more efficient characterization of the elastic symmetries of plane tensors can be provided by using the polar formalism [72,73,74]. In particular the orthotropy condition can be expressed in a more general form by abandoning the Cartesian representation of tensors, which is not based on invariant quantities. However, in order to keep same nomenclature of the reference paper [5] and for the sake of conciseness, investigation of the polar formalism is deferred to a future publication.

**Figure 1:**
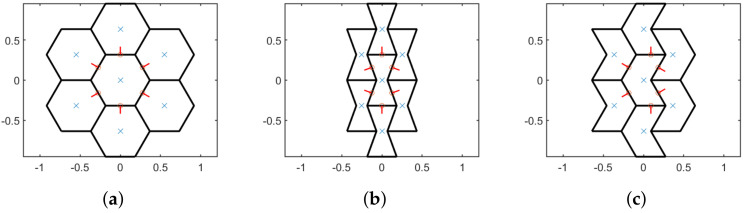
Seven blocks RVEs at larger scale (s=1): (**a**) regular (**b**) hourglass and (**c**) asymmetric.

The tiles interact among themselves through elastic joints centered on the middle point of the common edges; elastic linear translational and rotational stiffness are adopted. However, in order to apply the aforementioned methodology a reference volume element (RVE) must be detected: it is defined as the elementary volume element made of the minimal number of elements and joints sufficient to properly define the behavior of the material and it is the only one that preserve the material symmetry in the homogenization procedure [75]. In these analyses a RVE made of 7 blocks is considered. This work focuses on three different particle hexagonal geometries: regular, hourglass and asymmetric. Those geometries have been described in previous works by the authors [65] and they show peculiar constitutive behaviors such as orthotetragonal, auxetic and chiral elastic properties, respectively. The geometries of the considered RVEs are depicted in Figure 1. For each RVE configuration three scales are analyzed, named s=1, 0.5, 0.25.

Due to the orthotetragonal constitutive model of regular hexagons $B_{reg}=0$ (0 here indicates a 4×2 matrix) so there is no coupling between normal and shear stresses/strains with curvatures/micro-couples and consequently the material is centrosymmetric. The non-zero matrices of the current geometry are listed in Table 1. As previously observed in [65] regular hexagons are such that no coupling between normal stresses and shear strains (tangential strains and longitudinal strains) occurs. In addition, a small Poisson effect is shown.

The constitutive matrices for the hourglass shape are listed in Table 1 except for the coupling matrix which is zero $B_{hour}=0$ (0 here indicates a 4×2 matrix). As in the previous case, the scale effect is shown by matrix D and as aforementioned this configuration shows an auxetic behavior (negative Poisson effect since $A_{1122}$ and $A_{1221}$ are negative). This class of materials is relatively new: the first thermodynamic model and its numerical solution to show the possibility of negative Poisson’s ratio in molecular materials is due to [76], while the first material with auxetic properties was made by [77] and the first review of materials and structures with these new elastic properties as well as the origin of the name they bare today was reported in [78]. Furthermore dynamic properties of these materials [79,80,81,82], in particular some aspects related to dispersion process [10], (i.e., band gap), have already been studied [83].

Finally, the constitutive matrix for the asymmetric shape is given in Table 2. In the present configuration a coupling between stresses/curvatures (microcouples/strains) is present. There is no Poisson effect shown by the present configuration and scale effect is provided by both matrices B and D.

Starting from the above constitutive matrices, the same for the classical Cauchy continuum can be obtained [43] as:

$$C=\left[\begin{array}{ccc} A_{1111} & A_{1122} & 0\\ A_{2211} & A_{2222} & 0\\ 0 & 0 & \frac{1}{2}\left[A_{1212}+A_{2121}\right]+A_{1221} \end{array}\right]$$

It is worth mentioning that Cauchy continuum does not present any scale effect as well as no micro-couples (micro-rotation ω=0 is not included in the formulation).

The rotary inertia depends on the shape and the size of the micro-elements. Considering the entire reference volume element used in the homogenization technique the inertia is calculated for the whole RVE and then it is divided by the RVE area $A_{RVE}$:

(11) $$J_{c}=\frac{J}{A_{RVE}},\;\mathrm{for}\;J=\int_{A}\left(x^{2}+y^{2}\right)\;dA$$

where *J* is the inertia of the single tile, whereas $J_{c}$ is the inertia of the whole RVE. As a consequence there are three different rotary inertias for the three shapes which are scale dependent. The rotary inertia values for all configurations are reported in Table 3.

## 4. Numerical Implementation

In order to solve the present differential problem a finite element framework is implemented in MATLAB environment. The validity of this continuum micropolar model is verified by comparing the results to a discrete model where particles are modeled as rigid with elastic interactions among them.

### 4.1. Continuum Model

The present implementation follows the approach presented in [65] where Q4 finite element with reduced integration are employed and a rectangular FE mesh of 32 × 32 elements has been used. To perform reduced integration the strain vector has to be reordered by separating strain terms which are fully integrated and the ones for which reduced integration is applied. Once the problem is solved in terms of displacements other quantities such as stresses and relative rotation have to be post computed [63,64].

The finite element method enforces an approximation through nodal kinematic parameters as:

(12) $$u=N\;d^{e}$$

where the kinematic displacement vector is ordered as:

(13) $$d^{eT}=\left[\begin{array}{ccccccccc} u_{1}^{1} & ... & u_{1}^{4} & u_{2}^{1} & ... & u_{2}^{4} & \omega^{1} & ... & \omega^{4} \end{array}\right]$$

each finite element exhibits 12 degrees of freedom (3 per node). The matrix of the shape functions takes the form:

(14) $$N=\left[\begin{array}{ccc} N & 0 & 0\\ 0 & N & 0\\ 0 & 0 & N \end{array}\right]$$

where N is the vector of the linear Lagrangian shape functions. Below energy quantities required by the Hamilton Principle are provided. The kinetic energy reads:

(15) $$\delta K=-\delta d^{eT}\int_{A}N^{⊤}mN\;dA\;{\ddot{d}}^{\;e}$$

Finally, mass matrix is given by:

(16) $$M^{e}=\int_{A}N^{⊤}mN\;dA$$

The internal work takes the form:

(17) $$\delta U=\delta d^{eT}h\int_{A}{\left(D\;N\right)}^{⊤}C\left(D\;N\right)\;dA\;d^{e}=\delta d^{eT}h\int_{A}B^{⊤}C\;B\;dAd^{e}$$

where B=DN, thus the element stiffness matrix is:

(18) $$K^{e}=\int_{A}B^{⊤}C\;B\;dA$$

which has to be integrated according to a 2×2 Gauss integration for the normal components as well as micro-couples, whereas reduced integration is applied on shear components.

### 4.2. Discrete Model

In order to verify the equivalent continuum micropolar model, a discrete model is carried out in ABAQUS where particles are modeled as rigid and elastic (spring) interfaces are considered among the particles. Normal $K_{11}$ and shear $K_{22}$ stiffnesses are considered in the following, thus these springs have to be reported according to each local reference system for each elastic joint. In order to have a rigid behavior of blocks a high elastic modulus with respect to the elastic springs is considered [65].

## 5. Simulations

In this section the free vibration problem of a rectangular panel is analyzed for the three microstructured geometries introduced in the previous section. The analyses are conducted in reference to the following units: μg, μm, μs respectively for mass, length and time quantities. The panel, of rectangular planform ($L_{x}$, $L_{y}$), is clamped at the base. Such panel is considered with $L_{y}=7.7$ μm fixed and the following variable heights as:

- regular: $L_{x}=6.6$ μm;
- hourglass: $L_{x}=5$ μm;
- asymmetric: $L_{x}=5.85$ μm.

Constant stiffness is set among the particles for every scale, $K_{11}=0.785$ mN/μm as normal stiffness and $K_{22}=K_{11}/2=0.3925$ mN/μm as shear stiffness. In all simulations the material density is considered constant as $\rho =10^{-6}$μg/μm${}^{3}$.

### 5.1. Regular Geometry

**Figure 2:**
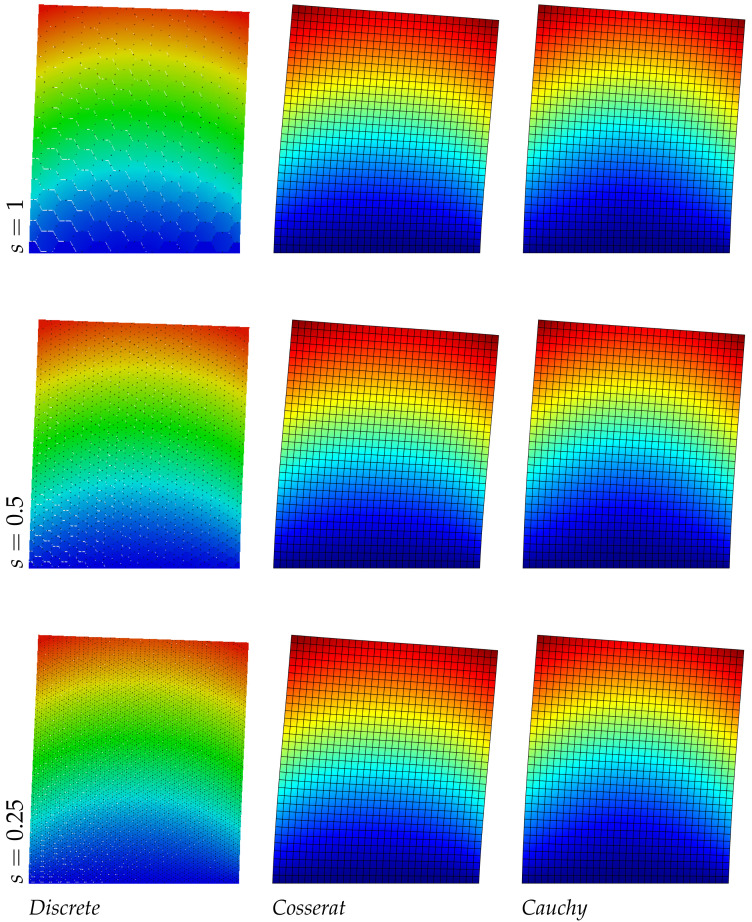
First natural vibration mode, regular geometry.

**Figure 3:**
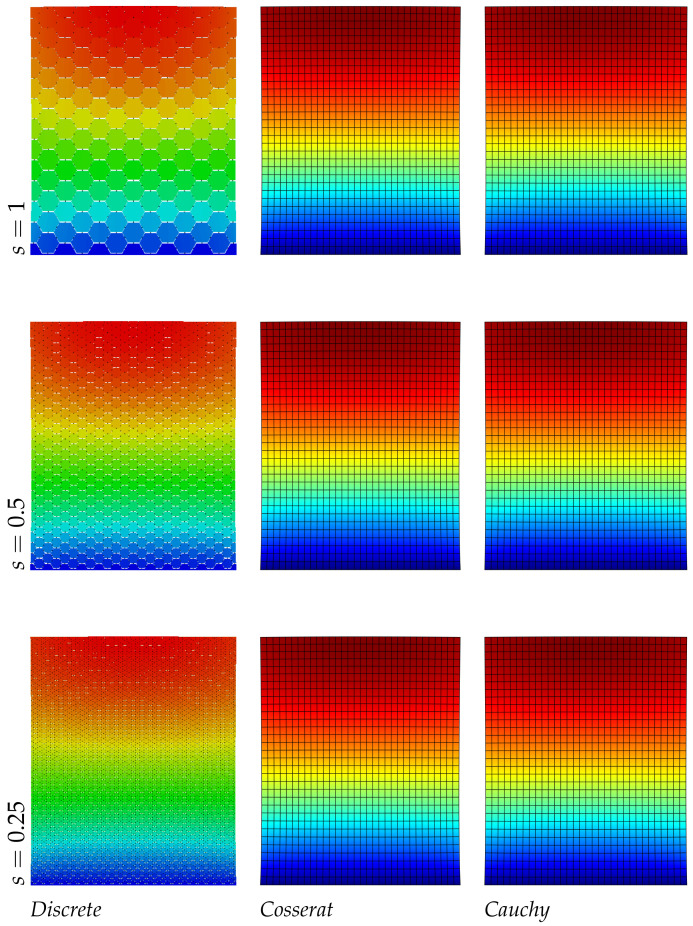
Second natural vibration mode, regular geometry.

**Figure 4:**
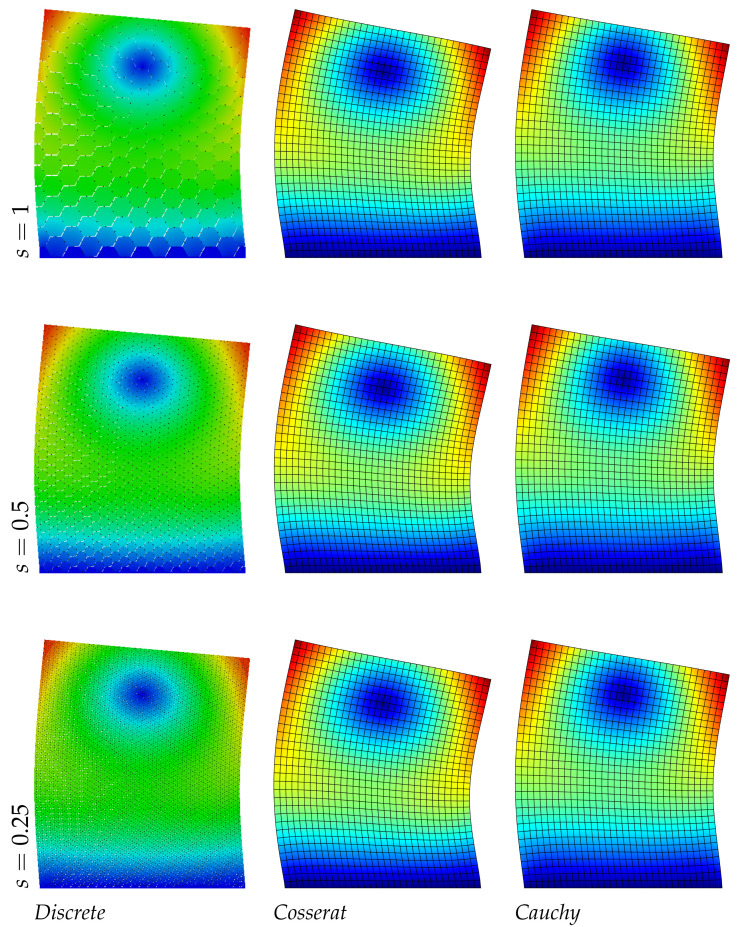
Third natural vibration mode, regular geometry.

The results of the panel made of regular hexagonal shapes for the first three modes are listed in Table 4. The same table reports the relative errors with respect to the discrete model of both micropolar and classical models. It is noted that the error in the classical model increases with the mode number, however for the present regular geometry (orthotetragonal constitutive behavior) the Cauchy model works quite well and similarly to the Cosserat one. Figure 2, Figure 3 and Figure 4 graphically represent the first three modes at three different scales for discrete, Cosserat and Cauchy models. First and third modes represent a bending mode with respect to *y* axis, whereas second mode is axial along *y* axis. Among all representations small differences are observed because of the orthotetragonal material considered.

### 5.2. Hourglass Geometry

**Figure 5:**
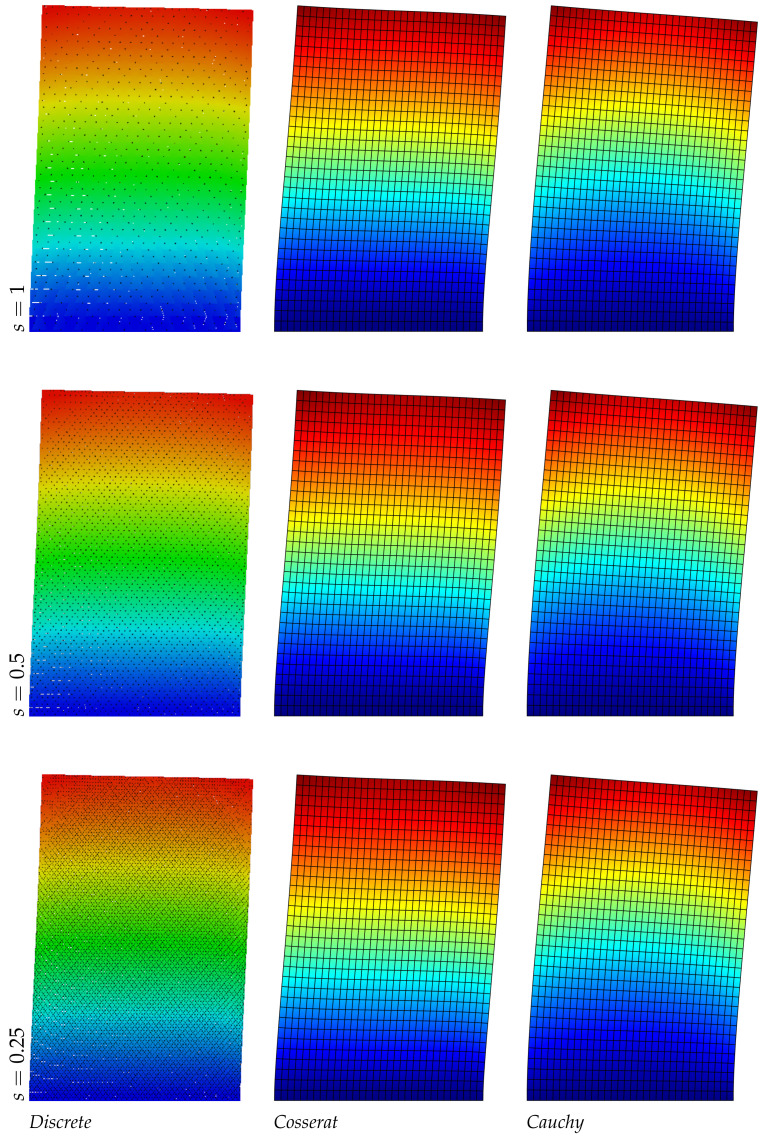
First natural vibration mode, hourglass geometry.

**Figure 6:**
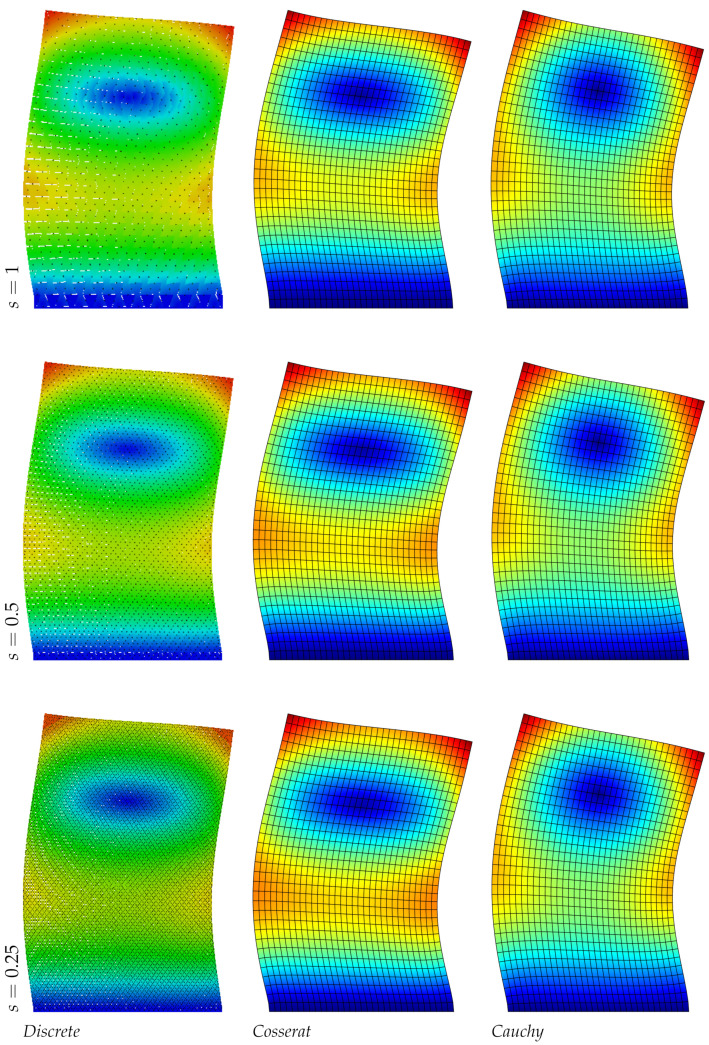
Second natural vibration mode, hourglass geometry.

**Figure 7:**
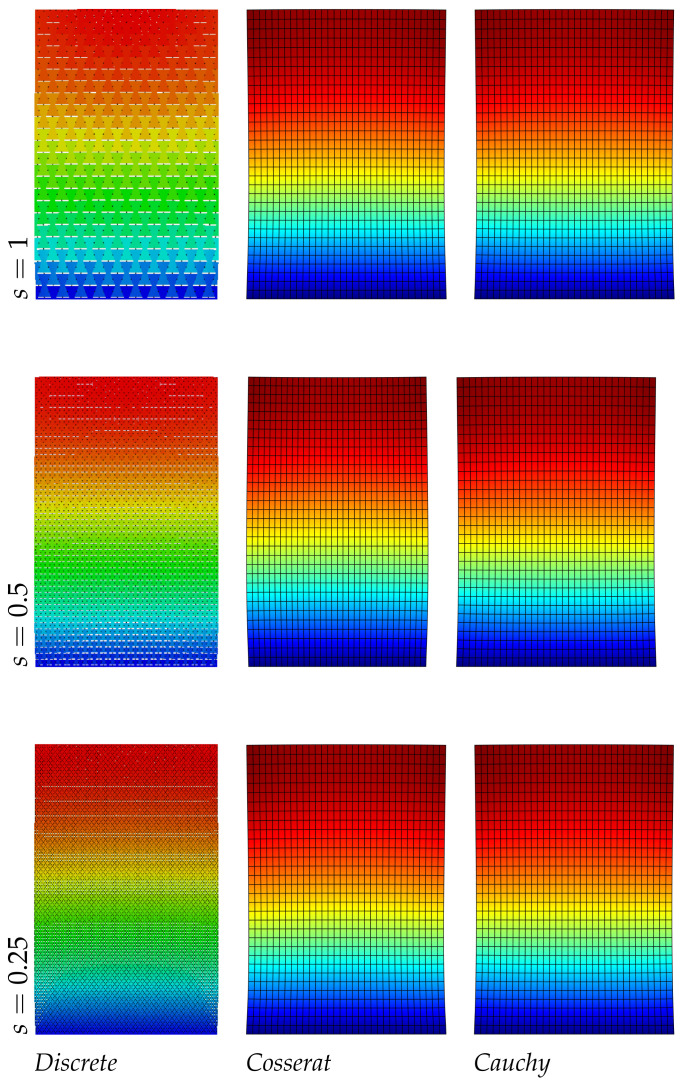
Third natural vibration mode, hourglass geometry.

In Table 5 the frequency values for the three models are reported: unlike the regular hexagonal microstructure, the differences on the frequency evaluation for the continua models is more marked and this is expected for the anisotropic nature of the material. For the Cosserat model the error is under the 1% for the smallest scale instead for the Cauchy model the maximum error is around the 45%, only the third mode, related to the axial vibrations, gives reliable results. Consequently, focusing the attention on the displacements fields (see Figure 5, Figure 6 and Figure 7) the micropolar model matches more with the discrete one for all modes. Lastly, it should be noted that the second and third vibration modes of the classical continuum are switched compared to the discrete system, therefore the second frequency value is greater than the third.

### 5.3. Asymmetric Geometry

**Figure 8:**
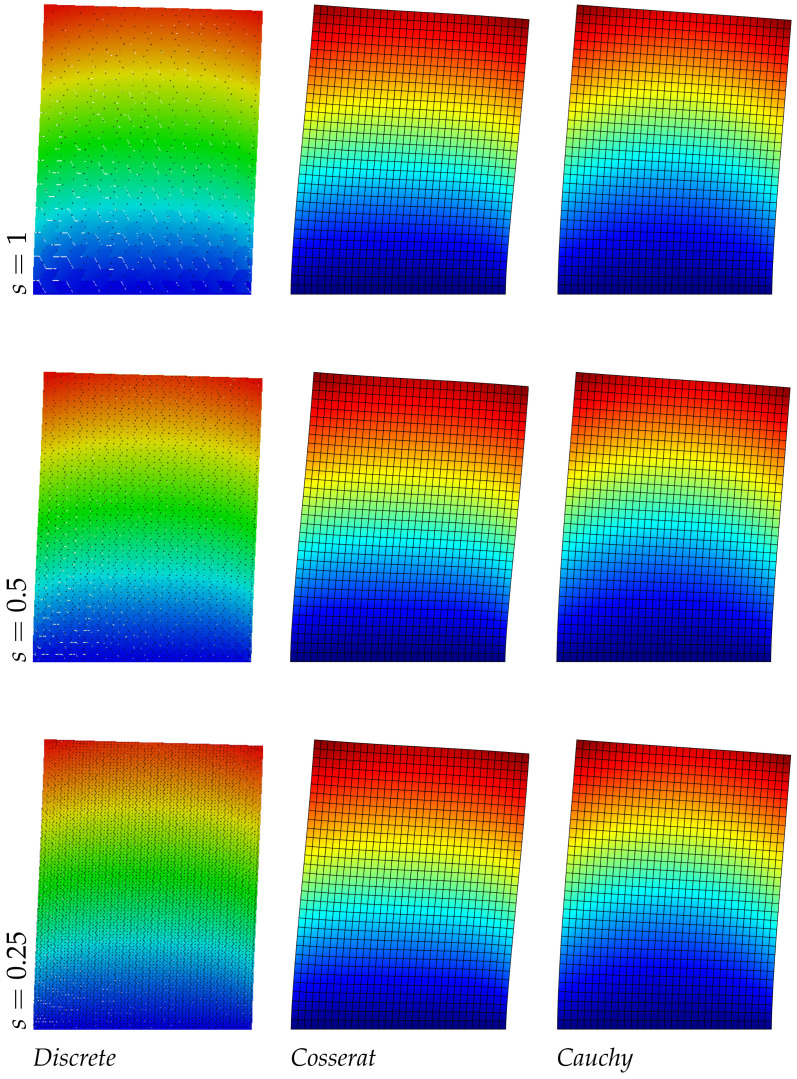
First natural vibration mode, asymmetric geometry.

**Figure 9:**
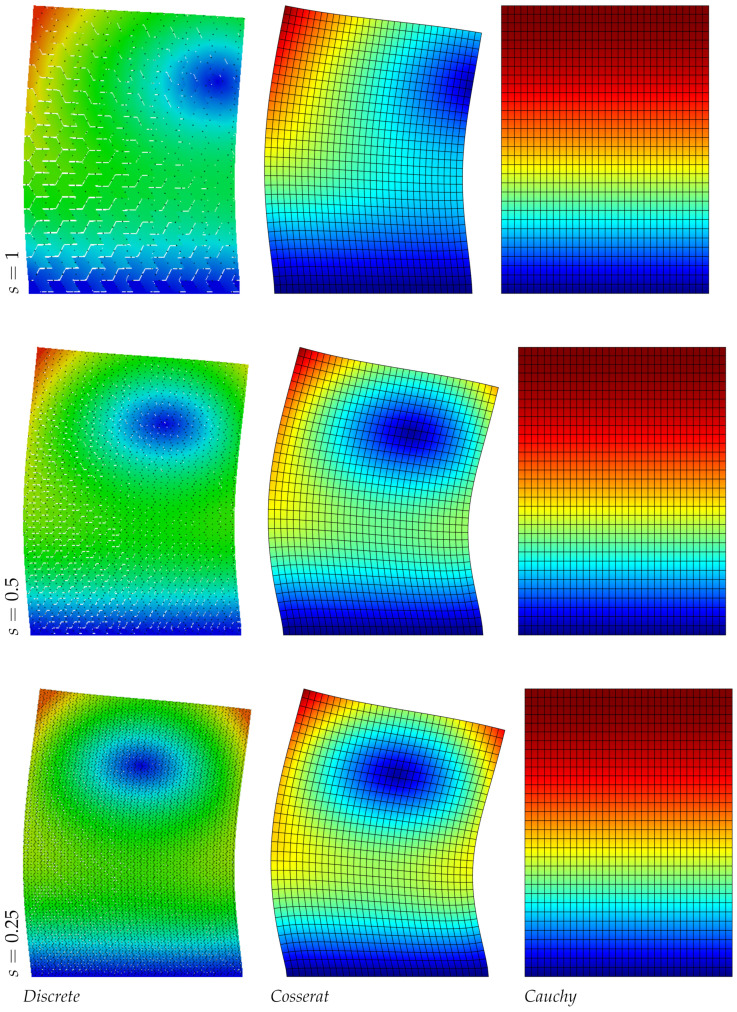
Second natural vibration mode, asymmetric geometry.

**Figure 10:**
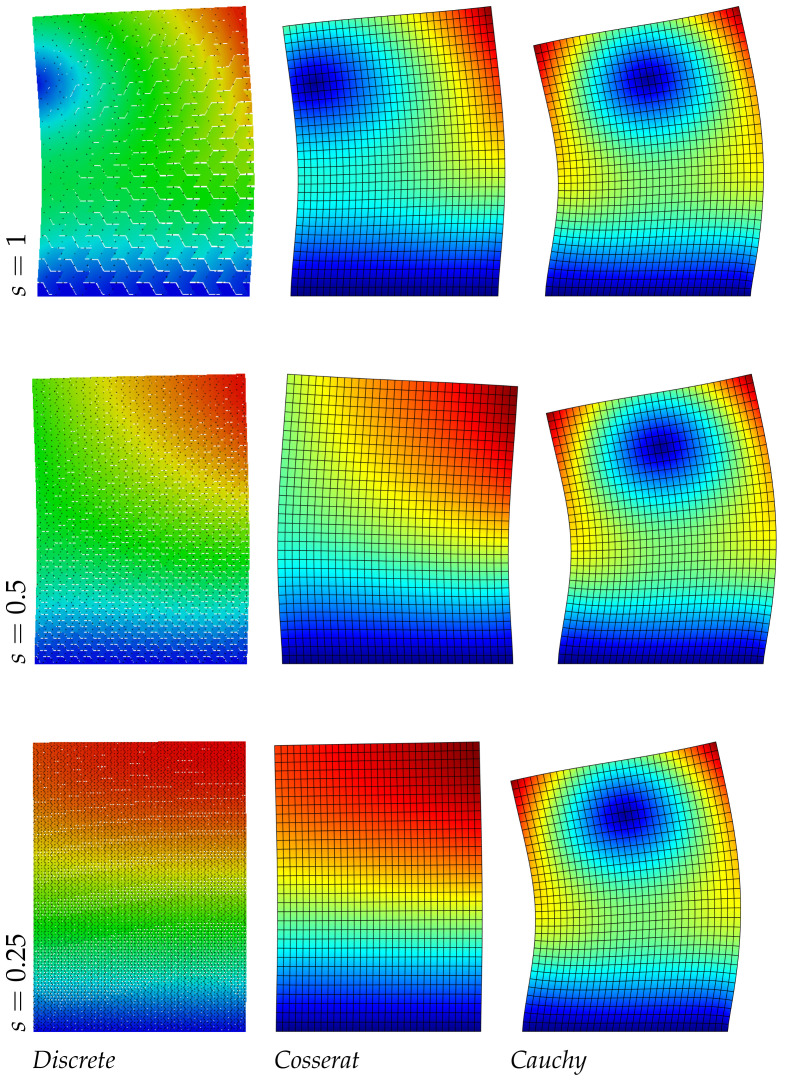
Third natural vibration mode, asymmetric geometry.

The results reported in Table 6 about the asymmetric microstructure confirm the previous trend, the Cosserat model is able to catch the frequency values of the discrete system with a good approximation (the maximum error is around 1%). The classical continuum is not able to predict the present material behavior. Moreover, a new aspect can be observed for the displacement fields in Figure 8, Figure 9 and Figure 10: the level curves of the second (Figure 9) and third (Figure 10) modes, change trend with the scale reduction due the asymmetry of the microstructure, differently from the regular and hourglass case and for all the three scales there is a good correspondence between the discrete and continuum Cosserat model. Obviously only the micropolar model can match with this trend because of his property of taking into account the internal length scale.

## 6. Conclusions

This work investigates the free vibration response of microstructured materials, with three different hexagonal shapes, in order to integrate the studies conducted previously [63,64,65] to have an enhanced and a complete overview about the mechanical behavior of these media and to highlight the advantages of a micropolar continuum representation. Moreover, the homogenization procedure provides reliable results also for the dynamic case and confirming the validity of the approach already tested for the static case [5,66,67]. The possibility of considering particle materials as continuous models considerably simplifies the modeling and the computational cost. It is worth noting as the best results in terms of frequency evaluation and displacement field representation are obtained for the smaller scale. This is the case of greatest interest because more computational burden for the discrete model is required, on the contrary, the computational cost of the equivalent micropolar model does not depend on the scale.

The three examined geometries present a different mechanical character: for regular hexagons an orthotropic behavior emerges and this is the only case in which the Cauchy model is able to give satisfactory results, and it is the same case where the scale reduction has less contribution; instead for the hourglass and asymmetric shapes, where the material assumes an anisotropic behavior and the scale dependence is more marked, only the micropolar continuum is able to match with the discrete model. What has already been done can be extended for different microstructure geometries, or for granular materials and for different constitutive laws at the microstructure scale.

## Figures and Tables

**Table 1:** Constitutive matrices for regular and hourglass blocks.

	Regular	Hourglass
	$A_{reg}$	$A_{hour}$
	$\left[\begin{array}{cccc} 1.1897 & 0.1700 & 0 & 0\\ 0.17 & 1.1897 & 0 & 0\\ 0 & 0 & 0.8498 & 0.1700\\ 0 & 0 & 0.1700 & 0.8498 \end{array}\right]$	$\left[\begin{array}{cccc} 0.5844 & -0.1261 & 0 & 0\\ -0.1261 & 2.5399 & 0 & 0\\ 0 & 0 & 1.9274 & -0.1261\\ 0 & 0 & -0.1261 & 0.3467 \end{array}\right]$
	$D_{reg}$	$D_{hour}$
s=1	$\left[\begin{array}{cc} 0.1082 & 0\\ 0 & 0.0882 \end{array}\right]$	$\left[\begin{array}{cc} 0.0407 & 0\\ 0 & 0.1969 \end{array}\right]$
s=0.5	$\left[\begin{array}{cc} 0.0270 & 0\\ 0 & 0.0221 \end{array}\right]$	$\left[\begin{array}{cc} 0.0102 & 0\\ 0 & 0.0492 \end{array}\right]$
s=0.25	$\left[\begin{array}{cc} 0.0068 & 0\\ 0 & 0.0055 \end{array}\right]$	$\left[\begin{array}{cc} 0.0025 & 0\\ 0 & 0.0123 \end{array}\right]$

**Table 2:** Constitutive matrices for asymmetric blocks.

Asymmetric	
$A_{asym}$	
$\left[\begin{array}{cccc} 0.7931 & 0 & 0 & 0\\ 0 & 1.7846 & 0 & 0\\ 0 & 0 & 1.2747 & 0\\ 0 & 0 & 0 & 0.5665 \end{array}\right]$	
$B_{asym}^{⊤}$	
s=1	$\left[\begin{array}{cccc} 0 & 0 & 0 & 0\\ 0 & 0.1244 & 0 & 0 \end{array}\right]$
s=0.5	$\left[\begin{array}{cccc} 0 & 0 & 0 & 0\\ 0 & 0.0622 & 0 & 0 \end{array}\right]$
s=0.25	$\left[\begin{array}{cccc} 0 & 0 & 0 & 0\\ 0 & 0.0311 & 0 & 0 \end{array}\right]$
$D_{asym}$	
s=1	$\left[\begin{array}{cc} 0.0655 & 0\\ 0 & 0.1516 \end{array}\right]$
s=0.5	$\left[\begin{array}{cc} 0.0164 & 0\\ 0 & 0.0379 \end{array}\right]$
s=0.25	$\left[\begin{array}{cc} 0.0041 & 0\\ 0 & 0.0095 \end{array}\right]$

**Table 3:** Rotational inertia for the RVE: *J* (μm^4^), $A_{RVE}$ (μm^2^), $J_{c}$ (μm^2^).

	RVE		
	$J\cdot 10^{-2}$	$A_{\mathrm{RVE}}$	$J_{c}\cdot 10^{-2}$
**Regular**			
s=1	35.73	1.392	25.68
s=0.5	2.230	0.348	6.406
s=0.25	0.141	0.087	1.621
**Hourglass**			
s=1	8.210	0.636	12.92
s=0.5	0.512	0.159	3.230
s=0.25	0.032	0.039	0.820
**Asymmetric**			
s=1	16.06	0.928	17.31
s=0.5	0.960	0.232	4.137
s=0.25	0.063	0.058	0.092

**Table 4:** Natural frequencies (MHz) for the regular shape.

Scale	Discrete	Cosserat	Error (%)	Cauchy	Error (%)
Mode 1					
s=1	14.78	14.07	−4.77	15.34	3.82
s=0.5	14.43	13.98	−3.10	15.34	6.37
s=0.25	14.22	13.94	−1.98	15.34	7.89
Mode 2					
s=1	35.98	35.12	−2.40	35.13	−2.38
s=0.5	35.64	35.11	−1.49	35.13	−1.45
s=0.25	35.45	35.11	−0.98	35.13	−0.93
Mode 3					
s=1	42.99	41.04	−4.56	49.24	14.52
s=0.5	42.15	41.10	−2.49	49.24	16.82
s=0.25	41.60	41.02	−1.41	49.24	18.36

**Table 5:** Natural frequencies (MHz) for the hourglass geometry.

Scale	Discrete	Cosserat	Error (%)	Cauchy	Error (%)
Mode 1					
s=1	13.03	13.55	3.97	17.06	30.95
s=0.5	12.86	12.94	0.62	17.06	32.64
s=0.25	12.76	12.70	−0.44	17.06	33.69
Mode 2					
s=1	39.88	43.39	8.77	56.83	42.46
s=0.5	39.22	40.39	2.98	56.83	44.88
s=0.25	38.79	39.04	0.63	56.83	46.47
Mode 3					
s=1	52.22	51.47	−1.43	51.48	−1.42
s=0.5	50.20	51.44	2.48	51.48	2.55
s=0.25	51.91	51.43	−0.92	51.48	−0.83

**Table 6:** Natural frequencies (MHz) for the asymmetric geometry.

Scale	Discrete	Cosserat	Error (%)	Cauchy	Error (%)
Mode 1					
s=1	14.50	14.44	−0.41	16.44	13.38
s=0.5	14.25	14.14	−0.76	16.44	15.38
s=0.25	14.11	14.03	−0.59	16.44	16.51
Mode 2					
s=1	42.48	42.93	1.06	43.38	2.11
s=0.5	42.41	42.39	−0.06	43.38	2.27
s=0.25	41.94	41.89	−0.11	43.38	3.41
Mode 3					
s=1	44.29	43.97	−0.72	52.40	18.30
s=0.5	43.55	43.45	−0.24	52.40	20.31
s=0.25	43.35	43.38	0.08	52.40	20.87

## Data Availability

Some or all data, models, or code that support the findings of this study are available from the corresponding author upon reasonable request.
